# Cloning and characterization of nitrate reductase gene in kelp *Saccharina japonica* (Laminariales, Phaeophyta)

**DOI:** 10.1186/s12870-023-04064-7

**Published:** 2023-02-06

**Authors:** Zhenghua Wang, Chunhui Wu, Peng Jiang

**Affiliations:** 1 CAS and Shandong Province Key Laboratory of Experimental Marine Biology, Center for Ocean Mega-Science, Institute of Oceanology, Chinese Academy of Sciences, Qingdao, 266071 China; 2 Laboratory for Marine Biology and Biotechnology, Qingdao National Laboratory for Marine Science and Technology, Qingdao, 266237 China; 3 University of Chinese Academy of Sciences, Beijing, 100049 China

**Keywords:** Function verification, Gene cloning, Kelp, Nitrate reductase, *Saccharina japonica*

## Abstract

**Background:**

Brown macroalgae dominate temperate coastal ecosystems, and their productivity is typically limited by nitrate availability. As an economically important kelp, *Saccharina japonica* is the most productive farmed seaweed and needs to be supplemented with sufficient nitrate throughout the cultivation process. However, molecular characterization of genes involved in nitrogen assimilation has not been conducted in brown macroalgae.

**Results:**

Here, we described the identification of the nitrate reductase (NR) gene from *S. japonica* (*SjNR*). Using two different cloning methods for *SjNR*, i.e. rapid amplification of cDNA ends (RACE) and cDNA cloning alone, a single fragment was obtained respectively. According to results of sequence analysis between these two fragments, the tentative coding sequence in two clones, *SjNR-L* and *SjNR-S*, were suggested to represent two transcripts of the single copy *SjNR*, and the ATG of *SjNR-S* was located inside the third exon of *SjNR-L*. In the 5′ upstream sequence of each transcript, promoter core elements, response elements, especially multiple N response elements which occurred in microalgal NR, were all predicted. Further sequence analysis revealed that both transcripts encoded all five domains conserved in eukaryotic plant NRs. RT-qPCR results showed that the transcription level of *SjNR* in juvenile sporophytes could be significantly induced by nitrate and inhibited by ammonium, which was in line with plant NRs. The recombinant SjNR-L and SjNR-S were all proved to have NR activity, suggesting that the single-copy gene *SjNR* might be regulated on transcription level based on alternative promoters and multiple transcriptional start sites. Moreover, both NADH and NADPH were found to be able to act as electron donors for SjNR alone, which is the first confirmation that brown algal NR has a NAD(P)H-bispecific form.

**Conclusion:**

These results will provide a scientific basis for understanding the N demand of kelp in various stages of cultivation and evaluating the environmental remediation potential of kelp in eutrophic sea areas.

**Supplementary Information:**

The online version contains supplementary material available at 10.1186/s12870-023-04064-7.

## Background

Nitrate reductase (NR) is a key enzyme in nitrogen (N) acquisition by plants, algae and fungi. NR can catalyze NAD(P)H reduction of nitrate to nitrite, which is a rate-limiting step for N acquisition in organisms [1]. NR is a soluble homodimer with an ~ 100 kDa polypeptide, which contains a molybdopterin domain, a heme domain, a FAD domain, a dimer interface domain and an extra binding domain for NAD(P)H in eukaryotes [1, 2]. Molybdopterin, heme and FAD domains are separated from one another by hinge 1 and hinge 2. Electrons are donated to the enzyme at the FAD by NAD(P)H, and the internal electron transport pathway is from FAD via heme to the molybdopterin. Finally, electrons are transferred to nitrate to complete the reduction process [1]. Typically, the transcription of NR gene is induced by nitrate, and repressed by ammonium [2, 3]. To be consistent with its physiological role, the NR activity is not only regulated by N sources but also by many environmental factors such as light, O_2_/CO_2_, pH and temperature, as well as plant metabolites and hormones [4, 5]. These regulations occur not only at the transcriptional level, but also at the post-transcriptional and translational levels [3, 6, 7].

Nitrate is a common form of inorganic N found in various marine ecosystems. In temperate coastal ecosystems, brown macroalgae dominate algal biomass and account for the majority of the primary production [8]. Productivity in these regions is typically limited by nitrate supply [9], and the degree of nutrient uptake and assimilation of brown macroalgae is higher than green or red macroalgae [10]. In addition to playing an important role in nearshore marine ecosystems, some species are also economically valuable for farming because of their ability to rapidly assimilate nutrients and grow quickly, among which *Saccharina japonica* Areschoug is the most productive. In China, the annual yields of *S. japonica* reached 10 million tons fresh weight in 2017, and it is still on the rise [11]. During the whole process of culture, including the stages for growth of both gametophytes and sporophytes of kelp, an adequate supply of additional nitrate is always required to ensure the rapid growth of *S. japonica* [12, 13]. In addition, in order to control the eutrophication and harmful algal blooms (HABs) in coastal areas, artificially cultured seaweeds have been widely proposed as an effective tool to balance and restore marine environments [14, 15]. As a species with the highest cultivated biomass, the removal of eutrophic elements from *S. japonica* has been evaluated in many marine areas [11, 16]. Therefore, the detailed study of NR gene in *S. japonica* (*SjNR*) will help to clarify the characteristics and regulatory mechanism of N metabolism in this economically important kelp, and provide a scientific basis for understanding the N demand of kelp in various stages of cultivation and evaluating the environmental remediation potential of kelp in eutrophic sea areas.

However, the existing studies on NR proteins of brown macroalgae were still relatively preliminary, and most of them were based on tissue homogenates to examine the enzyme activity of NR in vitro. Studies have shown that a variety of factors may affect the NR activity in brown macroalgae such as N sources [12, 17, 18], temperature [19], and light [8, 20]. In all tested species, such as *S. japonica*, *S. latissima* (synonym of *Laminaria saccharina*), *L. digitata*, and *Fucus serratus*, nitrate has been found to significantly increase the enzyme activity of NRs, and such effects were much higher in female gametophytes of *S. japonica* than in male [12]. For the brown algae living in the intertidal zone, it was found that their NR activity was not strongly induced by light or inhibited by ammonium compared with other brown algae living in the low intertidal-subtidal margin, which may be attributed to the obvious difference in main environmental stresses between two habitats [8, 18]. Additionally, the electron donor form of NR has been preliminarily investigated with tissue homogenates of brown algae *Fucus gardneri*. The results showed that NR activities could be detected by adding NADH or NADPH alone. However, the authors did not think that this brown algal NR belonged to NAD(P)H-bispecific form, but suggested that there might be isoenzymes including both independent NADH- and NADPH-specific forms, or, complex components in the homogenates may have converted NADPH to NADH [21]. In general, studies on the gene cloning and characterization of NR genes in brown algae are still missing so far.

In this study, our purpose was to clone the NR gene in *S. japonica* (*SjNR*), to characterize its transcriptional regulation, and to investigate its enzyme function and electron donor form to further understand nitrate assimilation mechanisms in this economically important brown alga. Moreover, in addition to its function in nitrate reduction, NR may also reduce chlorate to cytotoxic hypochlorite [22], allowing the survival of NR-deletion mutants in media containing hypochlorite. Therefore, NR has also been used as target genes for gene editing [23–25]. Since both genomic sequence data [26] and genetic transformation techniques for *S. japonica* were available [27, 28], *SjNR* is also expected to be used to develop gene editing techniques for this kelp.

## Methods

### Kelp strain and algal culture

The gametophyte clones of *Saccharina japonica* strain LDF01♀, LDF01♂ were donated by Engineer Xiaojie Li from Shandong Oriental Ocean Co., Ltd., and L049♀, L060♂ were kelp germplasm preserved in the laboratory. All the four gametophyte clones were originally isolated from cultivated *S. japonica* sporophytes decades ago. Gametophytes were maintained for vegetative growth in N/P-enriched autoclaved seawater (1.5 mM NaNO_3_, 0.17 mM NaH_2_PO_4_) at 10.0 ± 0.5 °C under a 10 h: 14 h (light period: dark period) photoperiod and illumination intensity of approximately 50 μmol photons m^− 2^ s^− 1^. The medium was renewed once a week. Young sporophytes were prepared from the cross between LDF01♀ and LDF01♂ according to a previously described protocol [27]. The gametophyte clonal cell clusters were ground gently using sterile glass slides to form short segments. After counting with a hemocytometer, approximately 1.0 × 10^6^ male or female gametophyte cells were mixture in seawater medium (0.286 mM NaNO_3_, 0.015 mM KH_2_PO_4_, 0.002 mM iron citrate, 0.5 μg/L vitamin B_12_), and cultured to induce sexual reproduction with an elevated irradiance to 100 μmol photons m^− 2^ s^− 1^ at 10.0 ± 0.5 °C under a 12 h: 12 h (light: dark) photoperiod. Germinated young sporophytes were transferred to the same conditions for the vegetative growth of gametophytes. All algal materials were cultured in an illumination incubator (Jiangnan, Ningbo, China).

### RNA extraction and cDNA synthesis

For algal RNA extraction, approximately 20 mg of gametophyte cell clusters or 200 mg of juvenile sporophytes (LDF01♀ × LDF01♂) about 2–3 cm in length were placed in a 1.5 mL EP tube, and centrifuged instantly to remove seawater. Total RNA of kelp was extracted according to the instruction of pBIOZOL Reagent (Bioflux, Beijing, China). The concentration and purity of RNA were determined by a NanoDrop2000 (Thermo Fisher Scientific, Waltham, MA, USA), and the integrity of RNA was evaluated by 1% (w/v) agarose gel electrophoresis. The cDNA synthesis was performed using a PrimeScript™ II 1st Strand cDNA Synthesis Kit (TaKaRa, Kusatsu, Shiga, Japan). Two or three repeats for gametophyte samples, and three repeats for sporophytes samples were conducted for RNA extraction and cDNA synthesis.

### Full-length cloning of *SjNR* cDNA

Using the amino acids (AAs) sequence encoded by the single copy NR gene of the model brown alga *Ectocarpus siliculosus* (GenBank: Esi0006_0124), a TBLASTN analysis (https://blast.ncbi.nlm.nih.gov/Blast.cgi) was performed with the genomic sequence of *S. japonica* (GenBank: MEHQ00000000.1). Aiming at the highly homologous sequence obtained, a pair of primers SjNR-F/R were designed to amplify the fragment of *SjNR* cDNA by PCR (Table 1), using the synthetic total cDNA of the female or male gametophytes as templates. PCR was carried out with a Programmable Master Cycler Personal Thermocycler (Thermal Cycler T960, Heal Force, Shanghai, China), following a protocol of 94 °C for 5 min, 35 cycles of 94 °C for 30 s, 58 °C for 30 s, and 72 °C for 2 min, and final 72 °C for 10 min. Each PCR product was cloned into the pGEM-T vector (Promega, Madison, Wisconsin, USA), then transformed to the Top10 competent cell of *Escherichia coli.* Plasmids were extracted and sent to Sangon Biotech Co. Ltd. (Shanghai, China) for sequencing.

**Table 1:** All primers used in this research

Amplification	Primers	Sequences (5′-3′)
cDNA fragment	SjNR-F	CTCCCACTCGGTCGCAAC
	SjNR-R	GCAAGCTGAACTTAGAAG
RACE	5-GSP^a^	GATTACGCCAAGCTTGAAGTTGAAGTGGCTGGTGCTGCTG
	3-GSP^a^	GATTACGCCAAGCTTCGCCAACGGCTACAGCAGCACCAG
	Long primer^b^	*CTAATACGACTCACTATAGGGCAAGCAGTGTATCAACGCAGAGT*
	Short primer^b^	*CTAATACGACTCACTATAGGGC*
qPCR	SjNR-qPCR-F	GGACGGGAGTGTGGGTGAAGGA
	SjNR-qPCR-R	CGTTGATGATGTAGGGGGGCTT
	SjUBA80-qPCR-F	AAGAAGACCTACACCAAGCCTA
	SjUBA80-qPCR-R	GTTTGGACGACACGGCACAGCG
CDS	pCold-SUMO-SjNR-L-F^c^	ggtaccctcgagggatccATGGCCTCAGGAATCCAATCG
	pCold-SUMO-SjNR-L-R^c^	gcagagattactctagaGAAGTTGAAGTGGCTGGTGC
	pCold-SUMO-SjNR-S-F^c^	ggtaccctcgagggatccATGGAGATGAGCGTGGAGTAC
	pCold-SUMO-SjNR-S-R^c^	gcagagattactctagaAAAGTTGAAGTGGCTGGTGC
	pIC9K-SjNR-F^d^	*agcttacgtagaattc*ATGGCCTCAGGAATCCAATCG
	pIC9K-SjNR-R^d^	*aattaattcgcggccgc*TTAGAAGTTGAAGTGGCTGGTGC
	5’AOX1^e^	*GACTGGTTCCAATTGACAAGC*
	3’AOX1^e^	*GCAAATGGCATTCTGACATCC*

^a^ Capital with underline indicates the homologous sequence with the end of pRACE vector for ligation by recombination

^b^ Capital italics indicates the primer provided by the SMARTer RACE 5′/3′ Kit

^c^ Lowercase indicates the homologous sequence with the end of pCold-SUMO vector for ligation by recombination

^d^ Lowercase italics indicates the homologous sequence with the end of pPIC9K vector for ligation by recombination

^e^ Capital italics with underline indicates the sequence of universal primer for pPIC9K vector

To determine the full-length of *SjNR* cDNA, the rapid amplification of cDNA ends (RACE) PCR was performed following the manufacturer’s instruction of a SMARTer RACE 5′/3′ Kit (Clontech, Mountain View, California, USA), with two primers 5-GSP and 3-GSP which were designed based on the sequence of *SjNR* cDNA fragment that was amplified by the primers SjNR-F/R. Because the primer 5-GSP was located downstream of 3-GSP, the full-length of *SjNR* cDNA might be obtained by assembling these two overlapped RACE-PCR products. The full-length genomic sequence of *SjNR* was not amplified, since it was shown to be more than 21 kb (supposed to be 21,214 bp) according to the genomic data [26]. Therefore, the deduced gene structure of *SjNR* was analyzed using the BLASTN (https://blast.ncbi.nlm.nih.gov/Blast.cgi) between the full-length of *SjNR* cDNA sequence and the homologous genomic sequence of *S. japonica*, and visualized using IBS 1.0 (http://ibs.biocuckoo.org). To determine the true initiation codon ATG, about 2 kb upstream sequence from each potential ATG was analyzed to detect the functional promoter elements, using an online tool plantCARE (http://bioinformatics.psb.ugent.be/webtools/plantcare/html).

### Phylogenetic analysis and alignment of deduced AA sequence of SjNR

The deduced AA sequence of SjNR was derived by using MEGA 6.0 program [29]. Phylogenetic analysis was performed with homologous AA sequences of NRs, including SjNR, reference sequences from other algae, moss, and higher plants. A Maximum-Likelihood (ML) phylogenetic tree was constructed using a LG + G model with MEGA 6.0 program, and the statistical support for individual branch was investigated by 1000 bootstrapping replicates [30]. Functional domains of SjNR were predicted by SMART (http://smart.embl-heidelberg.de/smart). DNAMAN V6 (Lynnon BioSoft, San Ramon, California, USA) was used for multiple sequences alignment and labeling functional domains. The protein molecular weight was calculated by Sequence Manipulation Suite (http://www.detaibio.com/sms2/index.html).

### Real-time quantitative PCR (qPCR) and *SjNR* transcript response with different N sources

Real-time quantitative PCR (qPCR) was performed to examine the mRNA relative abundance of *SjNR* gene with the Line-Gene 9600 Plus qPCR Detecting System (Bioer, Hangzhou, China) using TB Green fluorescence (TaKaRa, Kusatsu, Shiga, Japan). To reduce individual variations, each mRNA template was extracted with a mixture of three or four juvenile sporophytes. The PrimeScript™ RT Reagent Kit with gDNA Eraser (TaKaRa, Kusatsu, Shiga, Japan) was used to synthesize all cDNA templates. The ubiquitin fusion gene *UBA80* of *S. japonica* (*SjUBA80*) was employed to normalize the *SjNR* transcripts. Based on sequences of *SjNR* and *SjUBA80* cDNA fragments, two pairs of primers SjNR-qPCR-F/R, and SjUBA80-qPCR-F/R were designed for qPCR analysis (Table 1). The qPCR amplifications were carried out in a total volume of 20 μL containing 10 μL of 2× TB Premix Ex Taq™ II (TaKaRa, Kusatsu, Shiga, Japan), 0.8 μL (10 μM) of each primer, 2 μL of the diluted cDNA mix, and 6.4 μL deionized water. The qPCR amplification protocol was as follows: 40 cycles of 95 °C for 5 s, 60 °C for 30 s. The qPCR data was analyzed with the 2^-ΔΔCt^ method [31]. Three biological samples and repeated in triplicate were used in all qPCR experiments.

To detect the effect of N deprivation on *SjNR* transcript level, the juvenile *S. japonica* sporophytes were cultured for 6 days in fresh artificial seawater-based media without any nitrate additions. Just before N deprivation, samples were collected as controls (day 0), then the juvenile sporophytes were transferred to the N-free media with 0.015 mM KH_2_PO_4_, 0.002 mM iron citrate, and 0.5 μg/L vitamin B_12_ added, and samples were collected daily (day 1 ~ day 6) in the light period. Further, the response patterns of *SjNR* transcript level to nitrate or ammonium after N starvation were examined within one light period. At the beginning of the light period (light: dark = 10 h: 14 h), about 20 N-starved thalli were transferred to 200 mL fresh N-free media supplied by 2.86 mM either NO_3_^−^ (NaNO_3_) or NH_4_^+^ (NH_4_Cl) respectively. For each type of N-source group, three or four thalli were collected after 2, 4, 6, 8 h of N supplement, and the blank controls were sampled just before N supplement.

### Functional verification of SjNR in vitro

A pCold-SUMO-10His Prokaryotic Protein Expression Kit (HaiGene, Harbin, China) was employed to prepare the recombinant SjNR (rSjNR) protein for the functional verification in vitro. Two prime pairs, pCold-SUMO-SjNR-L-F/R and pCold-SUMO-SjNR-S-F/R, were designed to amplify the deduced complete coding sequence (CDS) regions for *SjNR-L* or *SjNR-S* (Table 1), from the previously constructed pGEM-T plasmids containing the cDNA fragments of *SjNR*. Using the In-Fusion® HD Cloning Kit (TaKaRa, Kusatsu, Shiga, Japan), the PCR products were cloned into the pCold-SUMO-10His vector which has been digested by *Bam*H I plus *Xba* I restriction endonucleases. According to the manufacturer’s instructions of the pCold-SUMO-10His Prokaryotic Protein Expression Kit, the constructed recombinant expression plasmids for SjNR, named pCold-SUMO-SjNR-L and pCold-SUMO-SjNR-S, were transformed to BL21 (DE3) competent cell of *E. coli* respectively, which were induced by Isopropyl β-D-thiogalactoside (IPTG) to express the recombinant proteins. In addition, an *E. coli* clone transformed with pCold-SUMO-10His was used as a negative control. The rSjNR proteins were purified with a Ni-NTA Resin Kit (HaiGene, Harbin, China), then eluted in the elution buffer [20 mM Tris-HCl (pH 7.9), 0.5 M NaCl, 50 mM Imidazole, 10% Glycerol], and stored at − 20 °C for further usage. Purity of the eluted protein was routinely documented by Coomassie Blue staining after SDS-PAGE [32], and the protein concentration was determined using a Bradford Protein Quantification Kit (Tiangen, Beijing, China).

The enzyme activity of rSjNR was determined according to previous descriptions with slight modifications [33, 34]. 100 μL of rSjNR solution was added to 900 μL of reaction solution which contained 100 mM HEPES (pH 7.6), 5 mM DTT, 10 mM MgCl_2_, 10 μM FAD, 10 μM sodium molybdate, 5 mM KNO_3_, and 0.2 mM NADH. The reaction was carried out at 24 °C for 10 min, then stopped by adding 125 μL of zinc acetate (0.5 M). 1 mL of Griess reagent [0.5 mL of 2% (w/v) sulfanilamide dissolved in 5% H_3_PO_4_, 0.5 mL of 0.2% (w/v) 1-Naphthyl ethylene diamine dihydrochloride (NEDD)] was added into the reaction mixture, following incubation for 10 min in dark at room temperature. Finally, the colorimetric determination of formed nitrite was conducted using a Multi-mode microplate reader (Tecan, Switzerland) at 546 nm. To calculate the half-saturation constants (K_m_) of rSjNR, the rSjNR activity was measured at gradient concentrations of KNO_3_ ranging from 0 to 20 mM, and the nonlinear regressions were performed using the curve fitting package of GraphPad Prism software version 5 (GraphPad Prism Software Inc., San Diego, California). In addition to the reaction solution with NADH, the replacement of NADPH was also examined to determine the form of rSjNR, and the reaction solutions with neither NADH nor NADPH were used as blank controls.

### Functional verification of SjNR in vivo

The recombinant expression of SjNR was conducted in *Pichia pastoris* with a pPIC9K vector. A pair of specific primers pPIC9K-SjNR-F/R was designed to amplify the deduced complete CDS region of *SjNR* as described above (Table 1). Using an In-Fusion® HD Cloning Kit (TaKaRa, Kusatsu, Shiga, Japan), the PCR products were cloned into the pPIC9K vector which has been digested by *Eco*R I plus *Not* I restriction endonucleases. The constructed vector pPIC9K-SjNR was digested by *Pme* I, then transformed into *P. pastoris* strain GS115 with the Gene pulser Xcell™ (Bio-Rad, California, USA) following described parameters and protocols [35]. Resistant transformants were obtained under the pressure of gradient concentrations of geneticin (0.1, 0.5, 1.0, 1.5, 2.0, 2.5, 3.0 mg/mL) [36]. The total genomic DNA for *P. pastoris* was extracted using a Yeast Genomic DNA Extraction Kit (Tiangen, Beijing, China). A pair of universal primer 5′ AOX1 and 3′ AOX1 was used to detect whether *SjNR* was inserted into the genome of *P. pastoris* (Table 1). The activities of rSjNR in *P. pastoris* were measured with a 96-well plate. 500 μL BMMY (Buffered Methanol-complex Medium) was transferred in a 1.5 mL EP tube, following the inoculation of one yeast transformant, the adding of 1% methanol to induce the expression of rSjNR, and 1% KNO_3_ as the substrate for the enzymatic reaction of NR. After incubation at 28 °C for 24 h, 50 μL cell supernatant plus 50 μL of Griess reagent was added to each well for reaction for 10 min in dark at room temperature, and the substrate color changes from colorless to magenta indicated the SjNR activity.

## Results

### cDNA cloning and characterization of *SjNR* gene

**Fig. 1:**
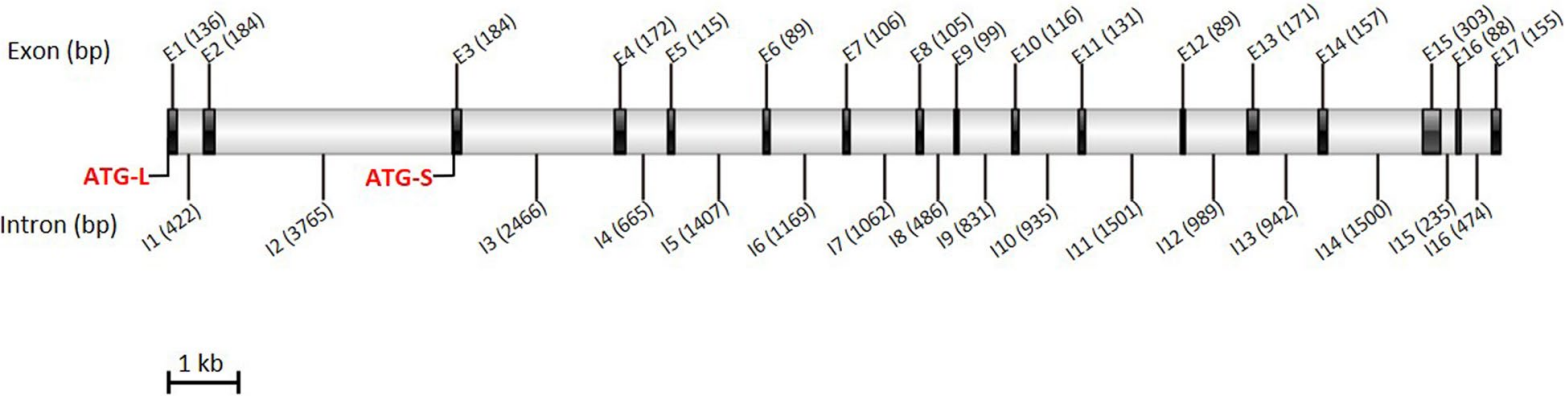
Structure of *SjNR* gene. Seventeen exons and sixteen introns were shown at relative positions. Characters E and I with a number represent each exon and intron, respectively, and number in brackets indicates the length of each unit. ATG-L indicates ATG of *SjNR-L* and ATG-S indicates ATG of *SjNR-S*

When the AA sequence encoded by the single copy NR gene of the model brown alga *Ectocarpus siliculosus* (Esi0006_0124) was used to perform the TBLASTN in the genomic data of *S. japonica* (MEHQ00000000.1), only one scaffold (JXRI01000482.1) with an identity value of more than 50% (83.18%) was obtained, suggesting that the homologous NR gene in *S. japonica* may be a single copy gene. Using primers SjNR-F/R and the synthetic total cDNA of LDF01♀ gametophyte as the template, a *SjNR* cDNA fragment containing the deduced 2466 bp CDS region was amplified (GenBank: OP184810). To clarify the gene structure of *SjNR*, the *SjNR* CDS sequence was used for BLASTN analysis with the genomic data of *S. japonica*. The results showed that the genomic sequence of *SjNR* contains 17 exons and 16 introns (Fig. 1). To determine the full-length of *SjNR* cDNA, 3′ RACE and 5′ RACE were carried out respectively. A 524 bp fragment was obtained from 3′ RACE, while after repeated attempts, only a 2244 bp fragment was yielded from 5′ RACE which was shorter than expected. Thus a shorter version of 2118 bp *SjNR* CDS was obtained by splicing these two overlapping sequences (GenBank: OP184811). For the sake of distinction, the shorter *SjNR* CDS was named *SjNR-S* and the longer *SjNR* CDS obtained previously was named *SjNR-L*. The corresponding start codon ATG was also named ATG-S and ATG-L respectively. Sequence alignment revealed that, ATG-S was located on the third exon of *SjNR-L*. Compared with *SjNR-L*, *SjNR-S* truncated only 348 bp at the 5′ end, and the rest sequences were completely consistent (Fig. 1).

To further determine whether these two ATGs have the possibility of acting as the start codon, about 2 kb upstream sequences from each potential ATG, which were named pSjNR-L and pSjNR-S respectively, were analyzed to detect the functional promoter elements. The results of PlantCARE analysis showed that in addition to core elements such as TATA-Box and CAAT-Box, other response elements for MeJA (TGACG-motif), drought (MBS), anoxic (GC-motif), zein (O2-site), light (ACE), and especially for N (GATA) which had been identified in the promoter region of NR gene from *Chlorella* [37], were also found in both sequences. Specific elements for light (G-box), meristem expression (CAT-box), abscisic acid (ABRE), and gibberellin (GARE-motif) were only found in pSjNR-L (Supplementary Fig. S1), and those elements for light (Sp1 and MRE), anaerobic (ARE), low temperature (LTR), defense or stress (TC-rich repeats) were only detected in pSjNR-S (Supplementary Fig. S2). These results suggest that there may be different transcription start sites in *SjNR* gene and they are regulated by different promoters.

In addition, multiple *SjNR* CDSs from different sexes and strains of gametophytes were analyzed. According to previous description for cloning *SjNR-L* cDNA from LDF01♀, homologous fragments containing the complete CDS region were amplified respectively from LDF01♂, L049♀, and L060♂ (GenBank: OP053687-OP053689) using primers SjNR-F/R. Sequences alignment of CDSs indicated multiple SNPs in different sexes and strains, and a three-base deletion in L060♂ (Supplementary Fig. S3). This was consistent with the single AA substitution or deletion in their AA sequence alignment (Supplementary Fig. S4), suggesting that there was obvious sequence polymorphism in the *SjNR* gene.

### Phylogenetic analysis and alignment of NR AA sequences

**Fig. 2:**
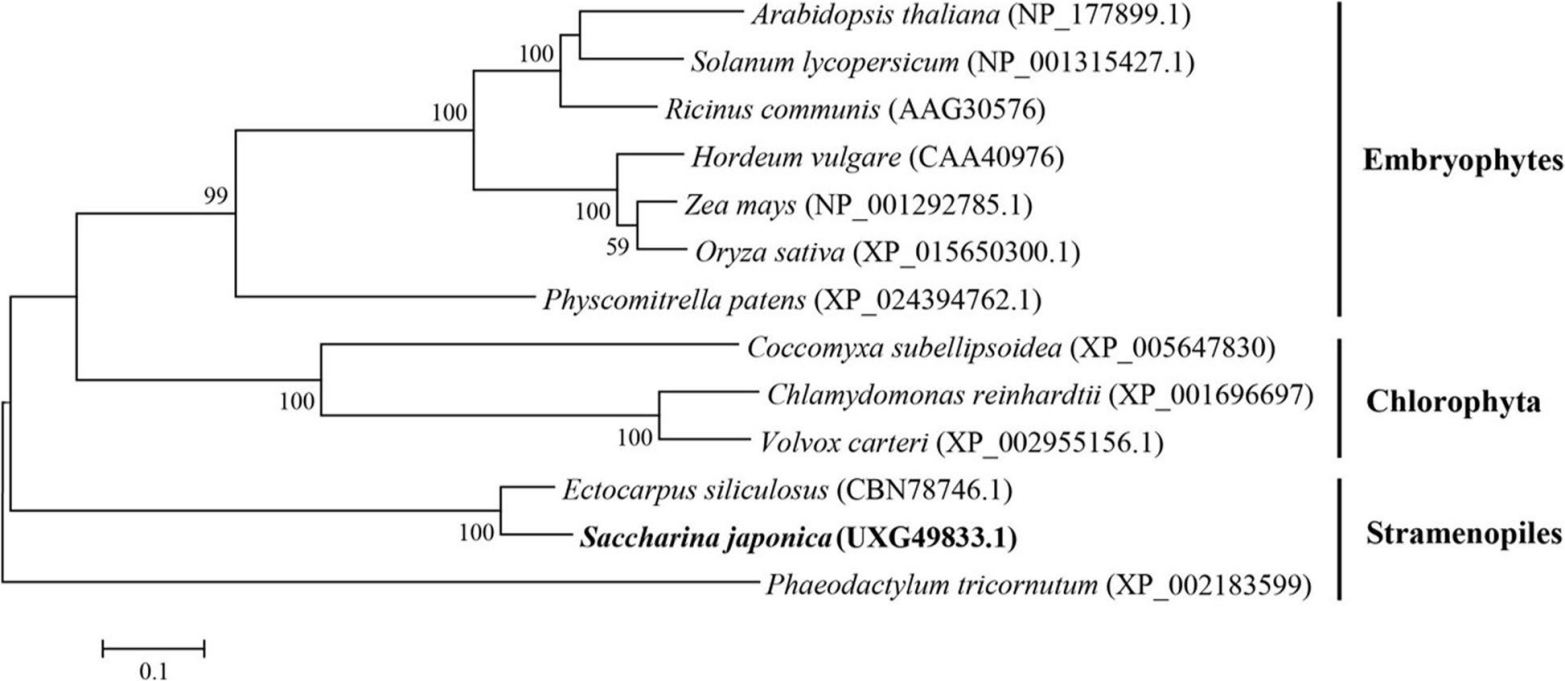
ML phylogenetic tree based on AA sequences of NRs. Numbers at the nodes indicate bootstrap values. GenBank accession numbers of all NR sequences are indicated. Sequence in bold was SjNR-L in this study

**Fig. 3:**
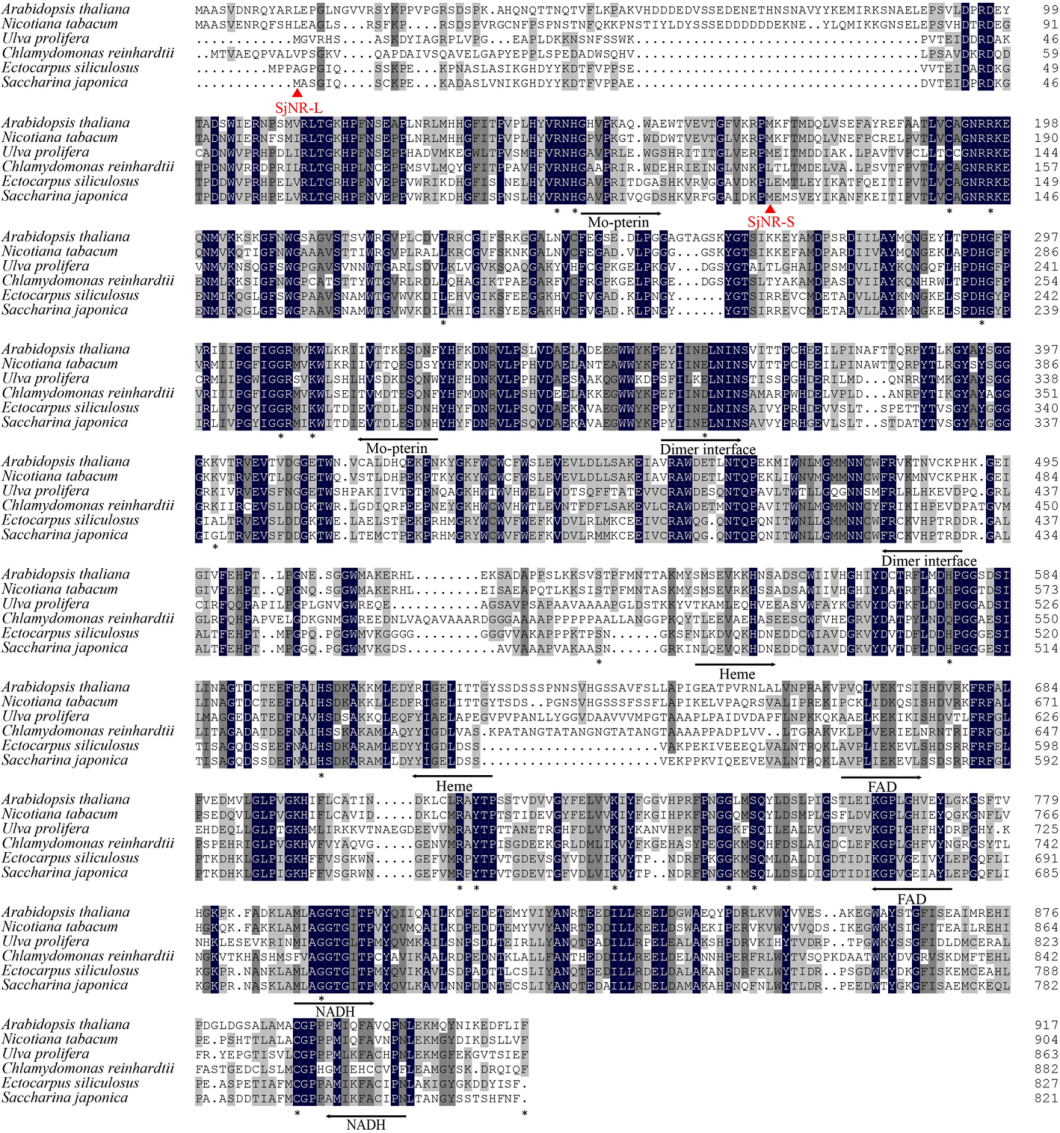
Alignment of NR AA sequences from higher plants, green algae and brown algae. Black arrows indicate the beginning and end of each functional domain: Mo-pterin for molybdopterin binding domain, Heme for heme binding domain, FAD for FAD binding domain, NADH for NADH binding domain. Asterisks below sequences denote AAs that had been identified as key invariant residues in AtNR from *Arabidopsis thaliana* [5]. Red arrows indicate the first AA for SjNR-L and SjNR-S. The six sequences aligned were NRs from *A. thaliana* (AAA32830.1), *Nicotiana tabacum* (XP_016462202.1), *Ulva prolifera* (ASV49153.1), *Chlamydomonas reinhardtii* (XP_001696697), and *Ectocarpus siliculosus* (CBN78746.1), as well as SjNR-L from *S. japonica* (UXG49833.1)

The ML phylogenetic tree was constructed with AA sequence of SjNR-L and 12 references from representative species. As shown in Fig. 2, SjNR-L was closest to NR from brown alga *Ectocarpus*, and then clustered with the NR of diatom *Phaeodactylum*, which also belonged to the Stramenopiles, but separated from NRs of green plants. SMART was used for the identification and annotation of domains, and the results showed that all five domains present in eukaryotic plant NRs could be found in both SjNR-L and SjNR-S, including molybdopterin binding domain, dimer interface domain, heme binding domain, FAD binding domain, and NADH binding domain. Compared with SjNR-L, the N-terminal of SjNR-S was short of 23 AAs. The molecular weights of SjNR-L and SjNR-S were calculated to be 91.80 kDa and 78.79 kDa by Sequence Manipulation Suite respectively. According to the alignment of NR AA sequences from higher plants, green algae, and brown algae (Fig. 3), it was observed that among the 21 key invariant residues in *Arabidopsis thaliana* NR (AtNR), Lys399 was the only different residue between brown algae and green plants which was substituted with Gly and Ala in *S. japonica* and *E. siliculosus* respectively.

### *SjNR* transcript response with different N sources

**Fig. 4:**
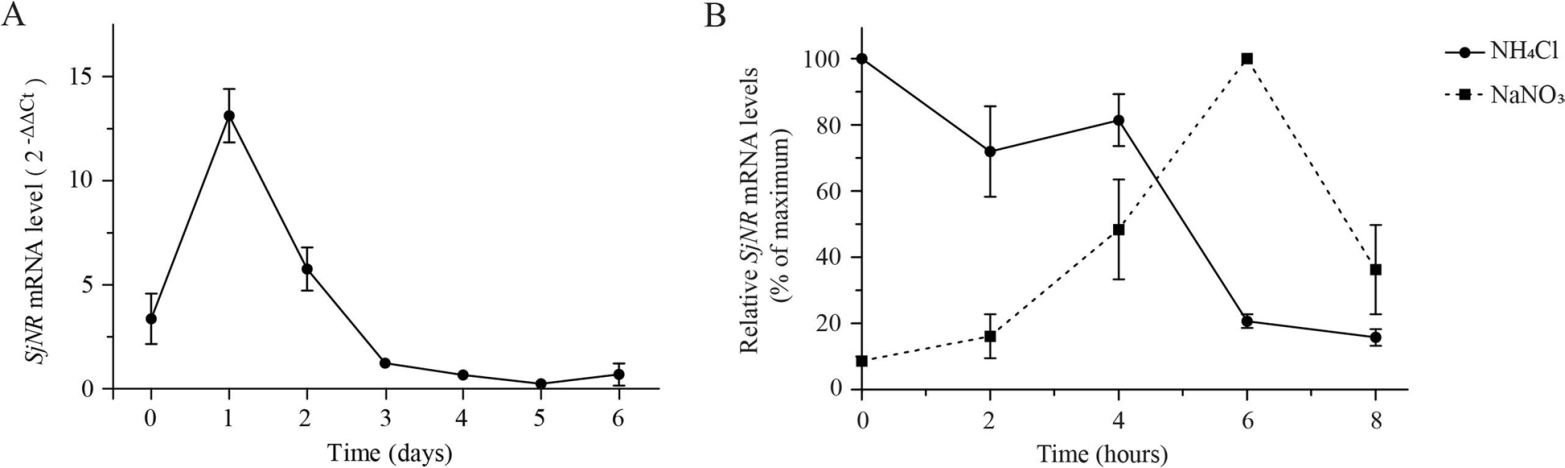
qPCR analysis of *SjNR* transcript level. **A** Evaluated *SjNR* transcript level under N deprivation. **B** Evaluated *SjNR* transcript levels to different N sources (NH_4_Cl or NaNO_3_). The *SjNR* transcript levels in two experiments were separately normalized by assuming that the highest level of *SjNR* transcript = 100%

To detect the effect of N deprivation on *SjNR* transcript level, the juvenile sporophytes of *S. japonica* were starved for six consecutive days, and qPCR was performed to examine the mRNA relative abundance of *SjNR* gene every day. The results showed that the *SjNR* transcript level increased after N starvation for 1 day, then dropped gradually and the mRNA level of *SjNR* reached the minimum value at the fifth day (Fig. 4A). As a result, the N-starved thalli were provided with different N sources after N starvation for 5 days, then sampled for qPCR analysis over 8 h. The responses of different N sources, i.e. nitrate and ammonium, on *SjNR* transcript levels were shown to be totally variant (Fig. 4B). After exposure to nitrate for 6 h, the mRNA level of *SjNR* reached the maximum point gradually, while decreased by about 70% at 8 h. On the contrary, the repression effect of ammonium on *SjNR* transcription was obvious, in which the transcript level decreased to a low level during the first 2 h. Although there was a rebound at the fourth hour, the curve dropped again and reached a lower level by the eighth hour.

### Functional verification of SjNR in vitro

**Fig. 5:**
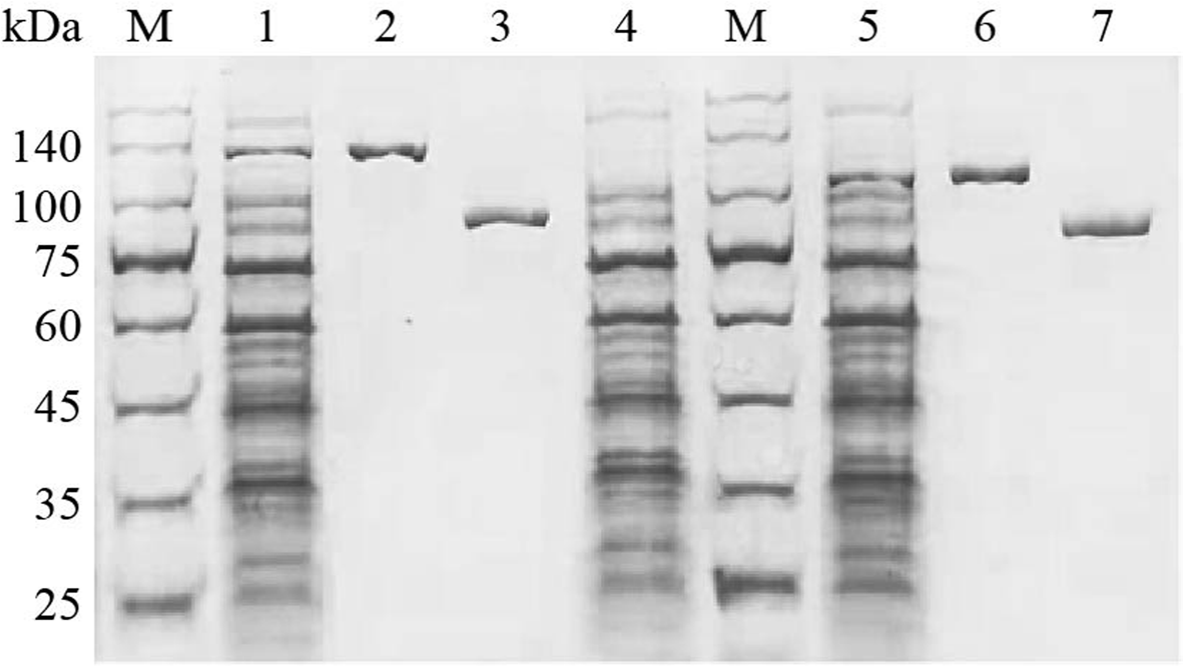
SDS-PAGE of rSjNR. M, protein marker; Lane 1, lysis of cells transformed with pCold-SUMO-SjNR-L; Lane 2, purified rSjNR-L with SUMO tag; Lane 3, purified rSjNR-L; Lane 4, negative control; Lane 5, lysis of cells transformed with pCold-SUMO-SjNR-S; Lane 6, purified rSjNR-S with SUMO tag; Lane 7, purified rSjNR-S. Full-length gels were presented in Supplementary Fig. S5

To analyze the function of SjNR in vitro, a commercial vector pCold-SUMO-10His was used for recombinant expression of SjNR-L and SjNR-S, in which the CS promoter was employed to drive gene transcriptions at low temperature, facilitate correct protein folding, and a SUMO tag was designed to further improve the solubility of recombinant proteins. Two plasmids with inserted CDS of SjNR-L or SjNR-S were constructed respectively, and were transformed to BL21 (DE3) competent cell of *E. coli*, which were induced by IPTG to express the recombinant proteins. The proteins rSjNR-L-SUMO and rSjNR-S-SUMO were purified with a Ni-NTA Resin Kit, then digested with SUMO protease to remove the SUMO tag. Purity of rSjNR-L and rSjNR-S were routinely documented by Coomassie Blue staining after SDS-PAGE (Fig. 5). The results showed that both purified rSjNR-L and rSjNR-S were stained as a single band with expected size, indicating the successful preparation.

**Fig. 6:**
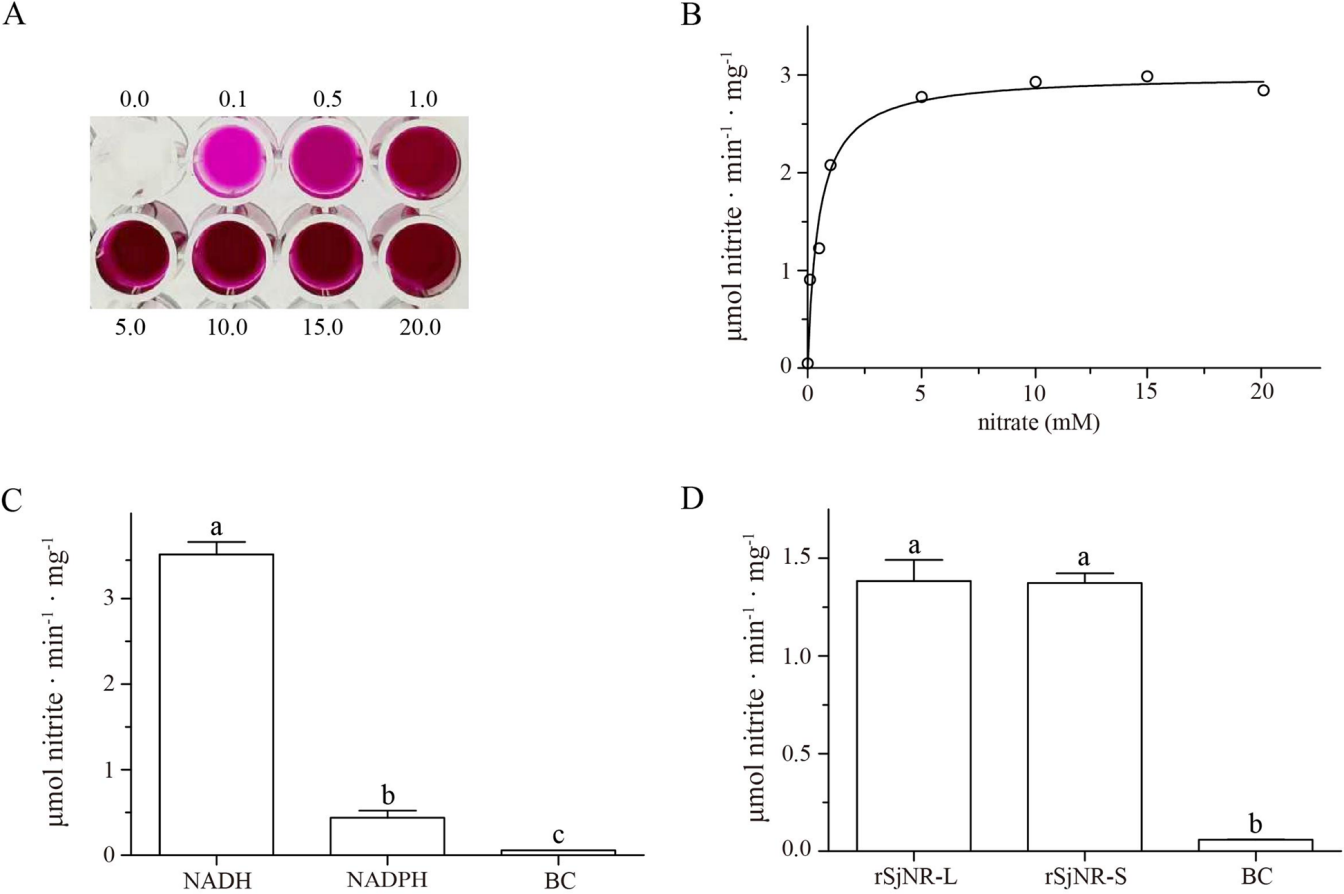
In vitro activity of rSjNR expressed in *E. coli*. **A** Coloration result of rSjNR-L under gradient concentrations of KNO_3_ (mM) with Griess reagent. **B** Kinetic curves of activity for rSjNR-L (Curves are fit to Michaelis-Menten models using nonlinear regression, as described in the text). **C** Evaluated activity of rSjNR-L with NADH or NADPH. **D** Evaluated activity of rSjNR-L and rSjNR-S. BC indicates blank control. Significant differences are indicated with lowercase (*P* < 0.05)

The activity of purified rSjNR-L and rSjNR-S was determined in vitro. Gradient concentrations of KNO_3_ ranging from 0 to 20 mM were used as the substrate to measure the activity of rSjNR-L. The results showed that all groups with added KNO_3_ could be dyed magenta by Griess reagent (Fig. 6A), indicating that rSjNR-L had NR activity. And the apparent half-saturation constant (Km) was 0.42 (± 0.059) mM for KNO_3_ (Fig. 6B). To detect the form of rSjNR-L, either NADH or NADPH was tested as the electron donor in the reaction solution, and the results showed that both of them could endow rSjNR-L with NR activity, while the replacement of NADH with NADPH reduced the rSjNR-L activity by about eight times (Fig. 6C). Furthermore, we found that the activity of rSjNR-S was lower than that of rSjNR-L, but there was no statistical difference (Fig. 6D).

### Functional verification of SjNR in vivo

**Fig. 7:**
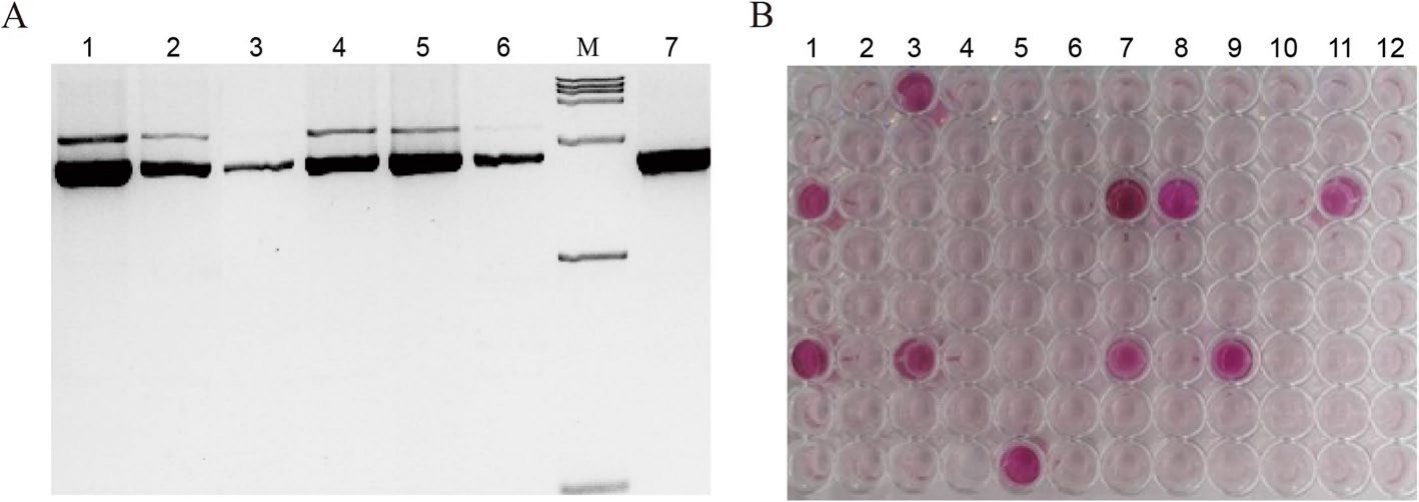
Detection of rSjNR expressed in *P. pastoris*. **A** PCR detection for positive transformants of *P. pastoris*. Lane 1, 2, 4, 5, positive transformants; Lane 3, 6, negative transformants; M, DNA marker; Lane 7, negative control. Full-length gels were presented in Supplementary Fig. S6. **B** Griess reagent dyeing of *P. pastoris*. Wells in magenta indicate positive transformants of *P. pastoris*. Column 1–11, positive transformants of *P. pastoris* with pIC9K-SjNR; Column 12, negative control

To verify the NR activity of rSjNR in vivo, *P. pastoris* which lacks endogenous NR were used as the expression system for SjNR-L heterologous expression and function examination. The full-length cDNA of *SjNR-L* was cloned into the expression vector pPIC9k-SjNR and transformed to *P. pastoris*. Resistant transformants were obtained under the pressure of geneticin, and the PCR detection has identified the integration of *SjNR-L* into the yeast genomes (Fig. 7A). Positive transformants were selected and cultivated separately. After induction with 1% methanol for the expression of rSjNR, 1% KNO_3_ was added as the substrate, and cell supernatant plus Griess reagent were used to detect the activity of rSjNR-L in vivo. The results showed that about 11% of the transformants media turned magenta (Fig. 7B), indicating that the recombinant SjNR-L in *P. pastoris* had the NR function in vivo.

## Discussion

In this study, the *SjNR* gene was obtained from the economically important kelp *S. japonica* by homologous cloning. The ML phylogenetic tree based on AA sequences showed that SjNR belongs to plant NRs, and all the five conserved domains in plant NRs were found in SjNR. The results of qPCR indicated the typical induction of SjNR transcript with nitrate and the repression with ammonium, and the NR activity of rSjNR were detected both in vitro and in vivo. Therefore, the NR gene of *S. japonica* has been successfully cloned and characterized in this study.

The alignment of AA sequences provided some new insights into the evolution of NR in plants. The results showed that the AA sequences in all NR domains were quite conserved across higher plants, green algae and brown algae (Fig. 3). Among the 21 key invariant residues in AtNR [5], a total of four residues were found to be mutated in algae. Three of the mutations, corresponding to Glu360, Ser534, and His577 in AtNR respectively, occurred only in green algae, while residues in these locations were exactly the same between brown algae and higher plants, implying that these three residues might undergo convergent evolution in these two groups. Lys399 in AtNR was found to be the only different residue between brown algae and green plants, and it also varied between *S. japonica* and *E. siliculosus*. It was reported that in AtNR, Glu360, Lys399 were located in the dimer interface domain for providing ionic bond at interface, His577 was located in the heme domain with the function of heme-Fe ligand, and Ser534 was located in Hinge I as the site of phosphorylation of NR [5]. When Ser534 in AtNR was replaced with Asp, the loss of inhibition of NR activity had been detected in an in vitro assay for regulation of NR [38]. It was speculated that the variation of these key residues between algae and higher plants was due to the selection pressure caused by different living environments.

The results of qPCR showed that the *SjNR* transcription could be significantly induced by nitrate and inhibited by ammonium in juvenile sporophytes, which was consistent with the typical performance of plant NRs. Brown macroalgae often dominate in both intertidal and subtidal zones, and abiotic stresses in the two habitats are obviously different. It was found that for *Laminaria digitata* living in low intertidal-subtidal margin, the NR activity could be significantly inhibited by ammonium and induced by light with a diel pattern [8], which was consistent with that of phytoplankton and higher plants [39, 40]. In contrast, the NR activities of *Fucus* spp. which occurs in intertidal zones were higher [41], and the regulation of NR activity with light and ammonium was apparently lacked [8]. In *Gracilaria chilensis*, another intertidal red alga, ammonium was even found to induce a temporary increase in NR activity [7]. A speculation was proposed that for intertidal algae, such particular NR regulation was suspected to be related to environmental characteristics in intertidal zones, such as briefer periods of immersion due to tidal cycles [8, 18]. *S. japonica* is typically a subtidal brown alga, and our results were consistent with those reports from other subtidal algae. In addition, as shown in Fig. 4A, the transcription level of *SjNR* in young sporophytes showed a transient increase after N starvation. Although this phenomenon is uncommon, it has also been reported in several algae, including green algae *Haematococcus pluvialis* and *Dunaliella salina* [42, 43], suggesting that this may be a compensation for plants to coping with N deficiency [41]. It was also found that the transcriptional activity of *SjNR* induced by nitrate decreased rapidly after reaching the peak value (Fig. 4B). This phenomenon was also observed in green algae, *Chlorella ellipsoidea* and *Ulva prolifera*, and explained to be a product inhibition of ammonium which was generated from final reduction of nitrate [3, 44]. It should be pointed out that gametophytes of kelp can also live independently, and the expression of *SjNR* gene in the gametophyte generation is worthy of further investigation in the future.

In this study, the recombinant SjNR was confirmed to have NR activity both in vitro and in yeast, and was determined as NAD(P)H-bispecific form. In general, there were three known forms of NR, NADH-specific (EC 1.7.1.1), NAD(P)H-bispecific (EC 1.7.1.2), and NADPH-specific (EC 1.7.1.3) [5]. All three forms of NR have been identified in bacteria, fungi, and higher plants. So far, only NADH-specific or NAD(P)H-bispecific forms have been found in algae. In general, NADH-specific form was dominant in algae, and has been widely identified in diatoms [45], haptophyte algae [46], red seaweeds [47], green microalgae [48], and green seaweed [49]. While NAD(P)H-bispecific form was only reported in red microalga [50], and green microalgae [51]. To our knowledge, this study is the first to identify the NR form in brown algae, and to report the NAD(P)H-bispecific form of NR in multicellular alga. In previous studies, in vitro experiments using homogenates from brown alga *Fucus gardneri* showed that NR activity could be detected by adding NADH or NADPH alone, and the activity of the former was 18 times higher than that of the latter [21]. The authors considered that NR isoenzymes including both NADH- and NADPH-specific might co-exist in *F. gardneri*, or added NADPH might be converted to NADH by dehydrogenase. A similar phenomenon was found later in red seaweed *Gracilaria chilensis* [52]. Our results might provide a new explanation for the findings in *F. gardneri* and *G. chilensis* that their NRs might also belong to the NAD(P)H-bispecific form. In addition, using *P. pastoris* without NR background as the expression host, it was confirmed that the heterologous rSjNR could utilize existing cofactors in yeast to obtain NR activity, indicating that this system could be used to verify the activity of exogenous NR in *P. pastoris*.

Interestingly, two homologous transcripts, namely SjNR-L and SjNR-S, were obtained by cDNA amplification and RACE respectively, suggesting that there were two potential initiation codon ATGs in the single-copy *SjNR* gene. In particular, promoters containing GATA elements were found in upstream sequences of both transcripts, which had been identified to response varieties of N sources for regulating the transcription of NR gene in green microalga [34]. Together with the fact that both rSjNR-L and rSjNR-S showed NR activity in vitro, here we hypothesized that the *SjNR* gene might contain multiple transcriptional start sites (TSSs) regulated by alternative promoters. For majority of eukaryotic genes, the presence of TSS clusters, variable initiation codons, or alternative promoters could increase the flexibility of gene expression at the transcriptional level, thus enhance the adaptation of species to environments [53–57]. Studies have shown that the NR expression was regulated at transcriptional, post-transcriptional, and translational levels [3, 6, 7]., responding to a variety of factors including N sources and light [5], At the transcriptional level, the usage of multiple TSSs or ATGs have been detected in plant NR genes. It was confirmed for the first time in *Chlorella* that the single-copy NR gene has two potential TSSs, and there were two putative ATGs within the first exon, which were driven by a single promoter to produce two NR transcripts, responsible for one induced expression and one constitutive expression respectively [58]. In *A. thaliana*, cap analysis of gene expression (CAGE) was used to detect genome-wide TSSs, and multiple TSSs have been found in NR genes after exposure to blue light [59]. In this study, the difference of *SjNR* gene was that its two ATGs were located in two adjacent exons with their own promoters, and the promoter of *SjNR-S* was located in the intron region of *SjNR-L* mRNA. Further efforts are still needed to verify the activity of two potential promoters with transgenic experiments, and to identify environmental factors that might determine their differentiated responses, such as different N sources and blue light, to which kelps are very sensitive [60, 61].

## Conclusions

In this study, the *SjNR* gene was cloned with two potential transcripts (*SjNR-L* and *SjNR-S*). For each transcript, all five domains conserved in eukaryotic plant NRs were encoded, and abundant N response elements which occurred in microalgal NR, were predicted in the 5′ upstream sequence. The transcription level of *SjNR* in juvenile sporophytes could be significantly induced by nitrate and inhibited by ammonium, which was in line with plant NRs. The recombinant SjNR-L and SjNR-S were all proved to have NR activity, suggesting that the single-copy gene *SjNR* might be regulated on transcription level based on alternative promoters and multiple TSSs. In addition, both NADH and NADPH were found to be able to act as electron donors for SjNR alone, which is the first confirmation that brown algal NR has a NAD(P)H-bispecific form. These results will provide a scientific basis for understanding the N demand of kelp in various stages of cultivation, and help to use *SjNR* as a target gene to develop gene editing techniques for *S. japonica*.

## Supplementary Information

**Additional file 1: Supplementary Fig. S1.** Regulatory elements in pSjNR-L. Their positions relative to SjNR-L + 1 start codon ATG are indicated. Vertical line points to the first base of each element.

**Additional file 2: Supplementary Fig. S2.** Regulatory elements in pSjNR-S. Their positions relative to SjNR-S + 1 start codon ATG are indicated. Vertical line points to the first base of each element.

**Additional file 3: Supplementary Fig. S3.** Multiple-sequence alignment of *SjNR-L* CDSs from strains LDF01♀, LDF01♂, L049♀, and L060♂. F: ♀; M: ♂.

**Additional file 4: Supplementary Fig. S4.** Alignment of SjNR AA sequences from strains LDF01♀, LDF01♂, L049♀, and L060♂. F: ♀; M: ♂.

**Additional file 5: Supplementary Fig. S5.** The original gel image of Fig. 5. Lane 1, 6, negative control; Lane 2, 7, protein marker; Lane 3, lysis of cells transformed with pCold-SUMO-SjNR-L; Lane 4, purified rSjNR-L with SUMO tag; Lane 5, purified rSjNR-L; Lane 8, lysis of cells transformed with pCold-SUMO-SjNR-S; Lane 9, purified rSjNR-S with SUMO tag; Lane 10, purified rSjNR-S.

**Additional file 6: Supplementary Fig. S6.** The original gel image including Fig. 7A. This gel image included two separate experimental gel diagrams, and the upper half was Fig. 7A. Lane 1–3, 6–7, 13, positive transformants; Lane 4, 9, DNA maker; Lane 5, 8, negative transformants; Lane 10, 11, negative control; Lane 12, invalid amplification.

## Data Availability

Sequencing datasets produced or investigated in this study are freely available at NCBI (OP184810; OP184811; OP053687; OP053688; OP053689).

## Abbreviations

AA: amino acid
RACE: rapid amplification of cDNA ends
CDS: coding sequence
IPTG: Isopropyl β-D-thiogalactoside
ML: maximum likelihood
N: nitrogen
NR: nitrate reductase
qPCR: real-time quantitative PCR
rSjNR: recombinant SjNR
*SjNR*: nitrate reductase gene of *Saccharina japonica*
TSS: transcriptional start site.

## References

[1] Campbell WH (2001). Structure and function of eukaryotic NAD(P)H:nitrate reductase. Cell Mol Life Sci.

[2] Solomonson LP, Barber MJ (1990). Assimilatory nitrate reductase: functional properties and regulation. Annu Rev Plant Physiol Plant Mol Biol.

[3] Wang P, Wang YQ, Geng DG, Li WB, Sun YR (2003). Molecular cloning and characterization of a cDNA encoding nitrate reductase from Chlorella ellipsoidea (Chlorophyta). J Appl Phycol.

[4] Mohr H, Neininger A, Seith B (1992). Control of nitrate reductase and nitrite reductase gene expression by light, nitrate and a plastidic factor. Botanica Acta.

[5] Campbell WH (1999). Nitrate reductase structure, function and regulation: bridging the gap between biochemistry and physiology. Annu Rev Plant Physiol Plant Mol Biol.

[6] Nussaume L, Vincentz M, Meyer C, Boutin JP, Caboche M (1995). Post-transcriptional regulation of nitrate reductase by light is abolished by an N-terminal deletion. Plant Cell.

[7] Chow F, De Oliveira MC (2008). Rapid and slow modulation of nitrate reductase activity in the red macroalga Gracilaria chilensis (Gracilariales, Rhodophyta): influence of different nitrogen sources. J Appl Phycol.

[8] Young EB, Dring MJ, Berges JA (2007). Distinct patterns of nitrate reductase activity in brown algae: light and ammonium sensitivity in Laminaria digitata is absent in Fucus species. J Phycol.

[9] Dugdale RC (1967). Nutrient limitation in the sea: dynamics, identification and significance. Limnol Oceanogr.

[10] Song M, Pham HD, Seon J, Woo HC (2015). Overview of anaerobic digestion process for biofuels production from marine.Macroalgae: a developmental perspective on brown algae. Korean J Chem Eng.

[11] Hu ZM, Shan TF, Zhang J, Zhang QS, Critchley AT, Choi HG, Yotsukura N, Liu FL, Duan DL (2021). Kelp aquaculture in China: a retrospective and future prospects. Rev Aquacult.

[12] Liu F, Pang SJ, Gao SQ (2016). Growth performance of unialgal gametophytes of the brown alga Saccharina japonica in mass culture conditions. J Appl Phycol.

[13] Wang YT, Xu D, Fan X, Zhang XW, Ye NH, Wang WQ, Mao YZ, Mou SL, Cao SN (2013). Variation of photosynthetic performance, nutrient uptake, and elemental composition of different generations and different thallus parts of Saccharina japonica. J Appl Phycol.

[14] Seghetta M, TøRring D, Bruhn A, Thomsen M (2016). Bioextraction potential of seaweed in Denmark — an instrument for circular nutrient management. Sci Total Environ.

[15] Kim JK, Yarish C, Hwang EK, Park M, Kim Y (2017). Seaweed aquaculture: cultivation technologies, challenges and its ecosystem services. Algae..

[16] Xiao X, Agusti S, Lin F, Li K, Pan Y, Yu Y, Zheng Y, Wu J, Duarte CM (2017). Nutrient removal from Chinese coastal waters by large-scale seaweed aquaculture. Sci Rep.

[17] Wheeler WN, Weidner M (1983). Effects of external inorganic nitrogen concentration on metabolism, growth and activities of key carbon and nitrogen assimilatory enzymes of Laminaria saccharina (Phaeophyceae) in culture. J Phycol.

[18] Young EB, Berges JA, Dring MJ (2009). Physiological responses of intertidal marine brown algae to nitrogen deprivation and resupply of nitrate and ammonium. Physiol Plantarum.

[19] Davison IR, Davison JO (1987). The effect of growth temperature on enzyme activities in the brown alga Laminaria saccharina. Br Phycol J.

[20] Davison IR, Stewart WDP (1984). Studies on nitrate reductase activity in Laminaria digitata (Huds.) Lamour. II. The rôle of nitrate availability in the regulation of enzyme activity. J Exp Mar Biol Ecol.

[21] Hurd CL, Berges JA, Osborne J, Harrison PJ (1995). An in vitro nitrate reductase assay for marine macroalgae: optimization and characterization of the enzyme for Fucus gardneri (Phaeophyta). J Phycol.

[22] Wilkinson JQ, Crawford NM (1993). Identification and characterization of a chlorate-resistant mutant of Arabidopsis thaliana with mutations in both nitrate reductase structural genes NIA1 and NIA2. Mol Gen Genet.

[23] Wang QT, Lu YD, Xin Y, Wei L, Huang S, Xu J (2016). Genome editing of model oleaginous microalgae Nannochloropsis spp. by CRISPR/Cas9. Plant J.

[24] Naduthodi MIS, Mohanraju P, Südfeld C, D'Adamo S, Barbosa MJ, van der Oost J (2019). CRISPR-Cas ribonucleoprotein mediated homology-directed repair for efficient targeted genome editing in microalgae Nannochloropsis oceanica IMET1. Biotechnol Biofuels.

[25] Kim J, Chang KS, Lee S, Jin E (2021). Establishment of a genome editing tool using CRISPR-Cas9 in Chlorella vulgaris UTEX395. Int J Mol Sci.

[26] Ye NH, Zhang XW, Miao M, Fan X, Zheng Y, Xu D, Wang JF, Zhou L, Wang DS, Gao Y, Wang YT, Shi WY, Ji PF, Li DM, Guan Z, Shao CW, Zhuang ZM, Gao ZQ, Qi J, Zhao FQ (2015). Saccharina genomes provide novel insight into kelp biology. Nat Commn.

[27] Jiang P, Qin S, Tseng CK (2003). Expression of the lacZ reporter gene in sporophytes of the seaweed Laminaria japonica (Phaeophyceae) by gametophyte-targeted transformation. Plant Cell Rep.

[28] Qin S, Jiang P, Tseng CK (2005). Transforming kelp into a marine bioreactor. Trends Biotech.

[29] Tamura K, Stecher G, Peterson D, Filipski A, Kumar S (2013). MEGA6: molecular evolutionary genetics analysis version 6.0. Mol Biol Evol.

[30] Hall BG (2013). Building phylogenetic trees from molecular data with MEGA. Mol Biol Evol.

[31] Livak KJ, Schmittgen TD (2001). Analysis of relative gene expression data using real-time quantitative PCR and the 2(−Delta Delta C(T)) method. Methods.

[32] Gallagher S, Sasse J. Protein analysis by SDS-PAGE and detection by Coomassie blue or silver staining. Curr Protoc Pharmacol. 2001;(Appendix 3):3B. 10.1002/0471141755.pha03bs02. 10.1002/0471141755.pha03bs02 21971792

[33] Tsai C, Kaiser WM, Kaldenhoff R (2003). Molecular cloning and characterization of nitrate reductase from Ricinus communis L. heterologously expressed in Pichia pastoris. Planta.

[34] Wany A, Pathak PK, Gupta KJ, Gupta KJ (2020). Methods for measuring nitrate reductase, nitrite levels, and nitric oxide from plant tissues. Nitrogen metabolism in plants.

[35] Lin-Cereghino J, Wong WW, Xiong S, Giang W, Luong LT, Vu J, Johnson SD, Lin-Cereghino GP (2005). Condensed protocol for competent cell preparation and transformation of the methylotrophic yeast Pichia pastoris. Biotechniques.

[36] Sunga AJ, Tolstorukov I, Cregg JM (2008). Posttransformational vector amplification in the yeast Pichia pastoris. FEMS Yeast Res.

[37] Cannons CA, Shiflett SD (2001). Transcriptional regulation of the nitrate reductase gene in Chlorella vulgaris: identification of regulatory elements controlling expression. Curr Genet.

[38] Su W, Huber SC, Crawford NM (1996). Identification in vitro of a post-translational regulatory site in the hinge 1 region of Arabidopsis nitrate reductase. Plant Cell.

[39] Berges JA, Cochlan WP, Harrison PJ (1995). Laboratory and field responses of algal nitrate reductase to diel periodicity in irradiance, nitrate exhaustion, and the presence of ammonium. Mar Ecol Prog Ser.

[40] Crawford NM, Smith M, Bellissimo D, Davis RW (1988). Sequence and nitrate regulation of the Arabidopsis thaliana mRNA encoding nitrate reductase, a metalloflavoprotein with three functional domains. Proc Natl Acad Sci U S A.

[41] Young EB, Lavery PS, van Elven B, Dring MJ, Berges JA (2005). Nitrate reductase activity in macroalgae and its vertical distribution in macroalgal epiphytes of seagrasses. Mar Ecol Prog Ser.

[42] Hou LL, Liu F, Zang XN, Zhang XC, He BX, Ding YT, Song XW, Xiao DF, Wang HT (2017). Cloning and transcription analysis of the nitrate reductase gene from Haematococcus pluvialis. Biotechnol Lett.

[43] Li J, Xue L, Yan H, Wang L, Liu L, Lu Y, Xie H (2007). The nitrate reductase gene-switch: a system for regulated expression in transformed cells of Dunaliella salina. Gene.

[44] Guo Y, Wang HZ, Wu CH, Fu HH, Jiang P (2017). Cloning and characterization of nitrate reductase gene in Ulva prolifera (Ulvophyceae, Chlorophyta). J Phycol.

[45] Gao Y, Smith GJ, Alberte RS (2000). Temperature dependence of nitrate reductase activity in marine phytoplankton: biochemical analysis and ecological implications. J Phycol.

[46] Iwamoto K, Shiraiwa Y (2003). Characterization of NADH: nitrate reductase from the coccolithophorid Emiliania huxleyi (Lohman) hay & Mohler (Haptophyceae). Mar Biotechnol.

[47] Chow F, Pedersén M, Oliveira MC (2013). Modulation of nitrate reductase activity by photosynthetic electron transport chain and nitric oxide balance in the red macroalga Gracilaria chilensis (Gracilariales, Rhodophyta). J Appl Phycol.

[48] Solomonson LP, Howard WD (1981). Kinetic mechanism of assimilatory NADH: nitrate reductase from Chlorella. J Biol Chem.

[49] Lartigue J, Sherman TD (2006). A field study of nitrogen storage and nitrate reductase activity in the estuarine macroalgae Enteromorpha lingulata (Chlorophyceae) and Gelidium pusillum (Rhodophyceae). Estuar Coast.

[50] Rigano C (1971). Studies on nitrate reductase from Cyanidium caldarium. Arch Mikrobiol.

[51] Lopez-Ruiz A, Verbelen JP, Roldan JM, Diez J (1985). Nitrate reductase of green algae is localized in the pyrenoid. Plant Physiol.

[52] Chow F, Oliveira MC, Pedersén M (2004). In vitro assay and light regulation of nitrate reductase in red alga Gracilaria chilensis. J Plant Physiol.

[53] Schibler U, Sierra F (1987). Alternative promoters in developmental gene expression. Annu Rev Genet.

[54] Landry JR, Mager DL, Wilhelm BT (2003). Complex controls: the role of alternative promoters in mammalian genomes. Trends Genet.

[55] Davuluri RV, Suzuki Y, Sugano S, Plass C, Huang TH (2008). The functional consequences of alternative promoter use in mammalian genomes. Trends Genet.

[56] Mejía-Guerra M, Li W, Galeano NF, Vidal M, Gray J, Doseff AI, Grotewold E (2015). Core promoter plasticity between maize tissues and genotypes contrasts with predominance of sharp transcription initiation sites. Plant Cell.

[57] Ushijima T, Hanada K, Gotoh E, Yamori W, Kodama Y, Tanaka H, Kusano M, Fukushima A, Tokizawa M, Yamamoto YY, Tada Y, Suzuki Y, Matsushita T (2017). Light controls protein localization through phytochrome-mediated alternative promoter selection. Cell.

[58] Dawson HN, Pendleton LC, Solomonson LP, Cannons AC (1996). Cloning and characterization of the nitrate reductase-encoding gene from Chlorella vulgaris: structure and identification of transcription start points and initiator sequences. Gene.

[59] Kurihara Y, Makita Y, Kawashima M, Fujita T, Iwasaki S, Matsui M (2018). Transcripts from downstream alternative transcription start sites evade uORF-mediated inhibition of gene expression in Arabidopsis. Proc Natl Acad Sci U S A.

[60] Wang WJ, Sun XT, Wang GC, Xu P, Wang XY, Lin ZL, Wang FJ (2010). Effect of blue light on indoor seedling culture of Saccharina japonica (Phaeophyta). J Appl Phycol.

[61] Wang WJ, Wang FJ, Sun XT, Liu FL, Liang ZR (2013). Comparison of transcriptome under red and blue light culture of Saccharina japonica (Phaeophyceae). Planta..

