# Supplementary material for: Cloning and characterization of nitrate reductase gene in kelp Saccharina japonica (Laminariales, Phaeophyta)
Source: BMC Plant Biol. 2023 Feb 6;23:78. doi: 10.1186/s12870-023-04064-7 (PMC9901164; doi:10.1186/s12870-023-04064-7)
Supplement: Supplementary file 2 — Additional file 2: Supplementary Fig. S2. Regulatory elements in pSjNR-S. Their positions relative to SjNR-S + 1 start codon ATG are indicated. Vertical line points to the first base of each element. [file 12870_2023_4064_MOESM2_ESM.pptx]

## Slide 1
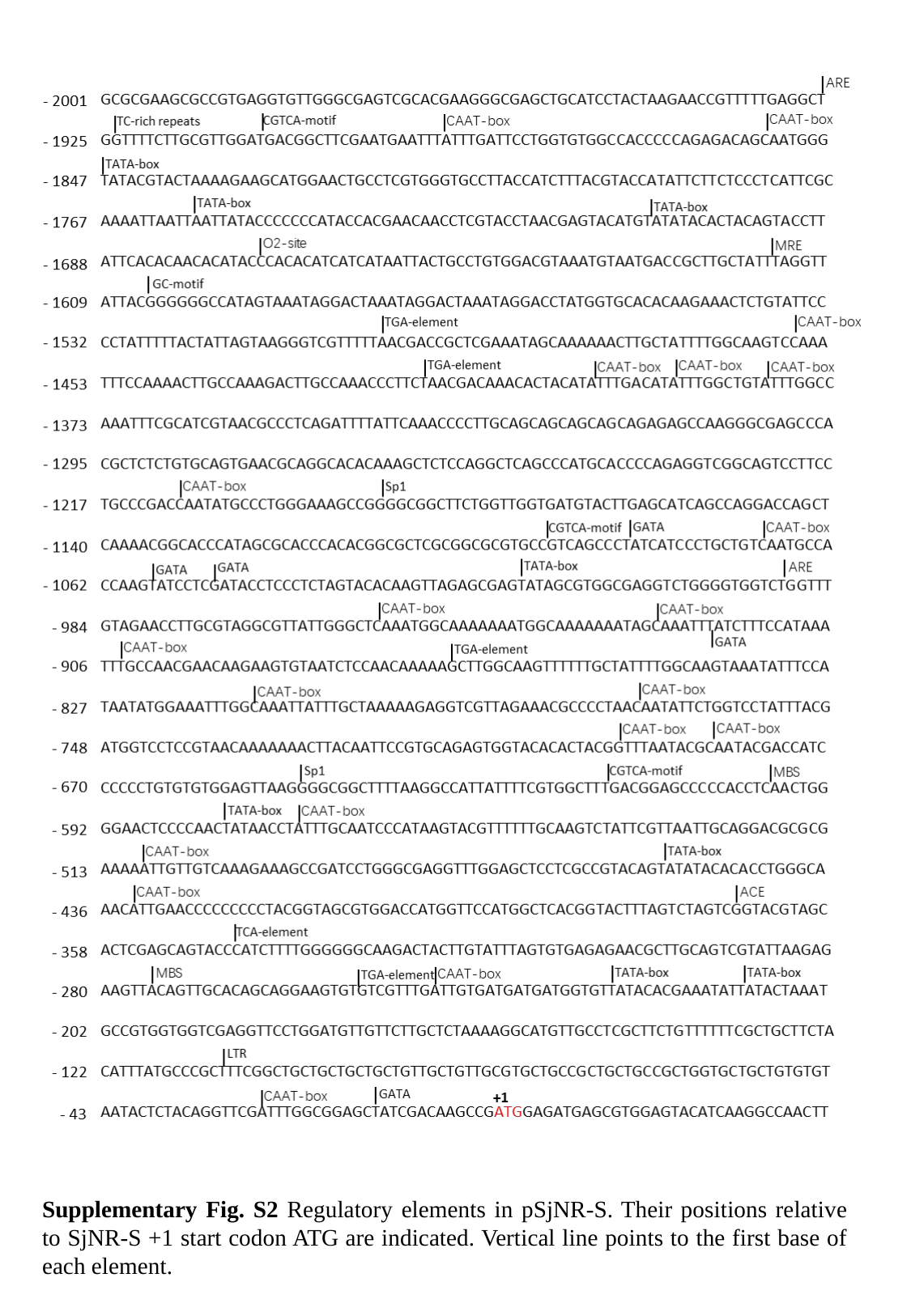

Supplementary Fig. S2 Regulatory elements in pSjNR-S. Their positions relative to SjNR-S +1 start codon ATG are indicated. Vertical line points to the first base of each element.
