# Supplementary material for: Cloning and characterization of nitrate reductase gene in kelp Saccharina japonica (Laminariales, Phaeophyta)
Source: BMC Plant Biol. 2023 Feb 6;23:78. doi: 10.1186/s12870-023-04064-7 (PMC9901164; doi:10.1186/s12870-023-04064-7)
Supplement: Supplementary file 3 — Additional file 3: Supplementary Fig. S3. Multiple-sequence alignment of SjNR-L CDSs from strains LDF01♀, LDF01♂, L049♀, and L060♂. F: ♀; M: ♂. [file 12870_2023_4064_MOESM3_ESM.pptx]

## Slide 1
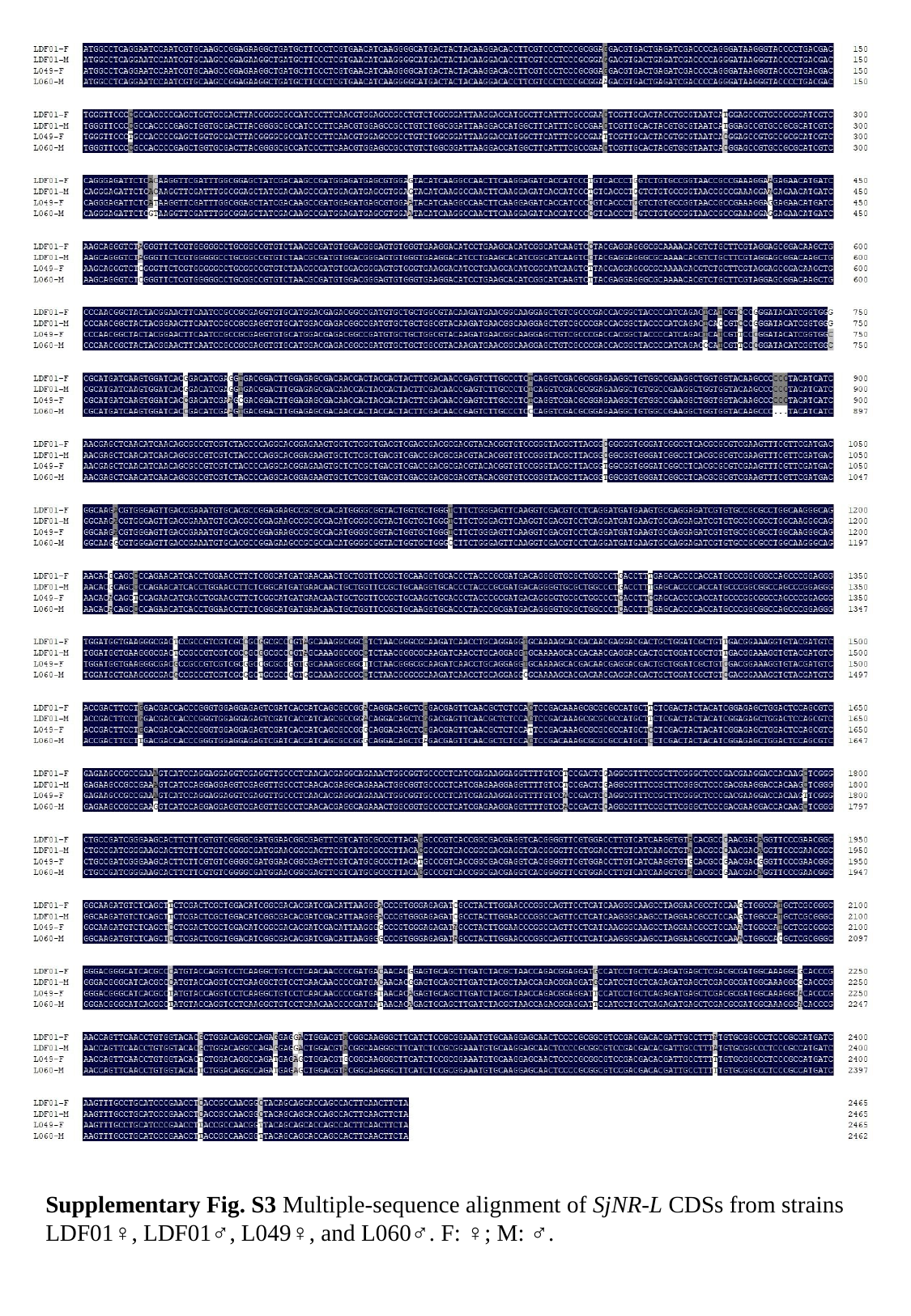

Supplementary Fig. S3 Multiple-sequence alignment of SjNR-L CDSs from strains LDF01♀, LDF01♂, L049♀, and L060♂. F: ♀; M: ♂.
