# Supplementary material for: Cloning and characterization of nitrate reductase gene in kelp Saccharina japonica (Laminariales, Phaeophyta)
Source: BMC Plant Biol. 2023 Feb 6;23:78. doi: 10.1186/s12870-023-04064-7 (PMC9901164; doi:10.1186/s12870-023-04064-7)
Supplement: Supplementary file 5 — Additional file 5: Supplementary Fig. S5. The original gel image of Fig. 5. Lane 1, 6, negative control; Lane 2, 7, protein marker; Lane 3, lysis of cells transformed with pCold-SUMO-SjNR-L; Lane 4, purified rSjNR-L with SUMO tag; Lane 5, purified rSjNR-L; Lane 8, lysis of cells transformed with pCold-SUMO-SjNR-S; Lane 9, purified rSjNR-S with SUMO tag; Lane 10, purified rSjNR-S. [file 12870_2023_4064_MOESM5_ESM.pptx]

## Slide 1
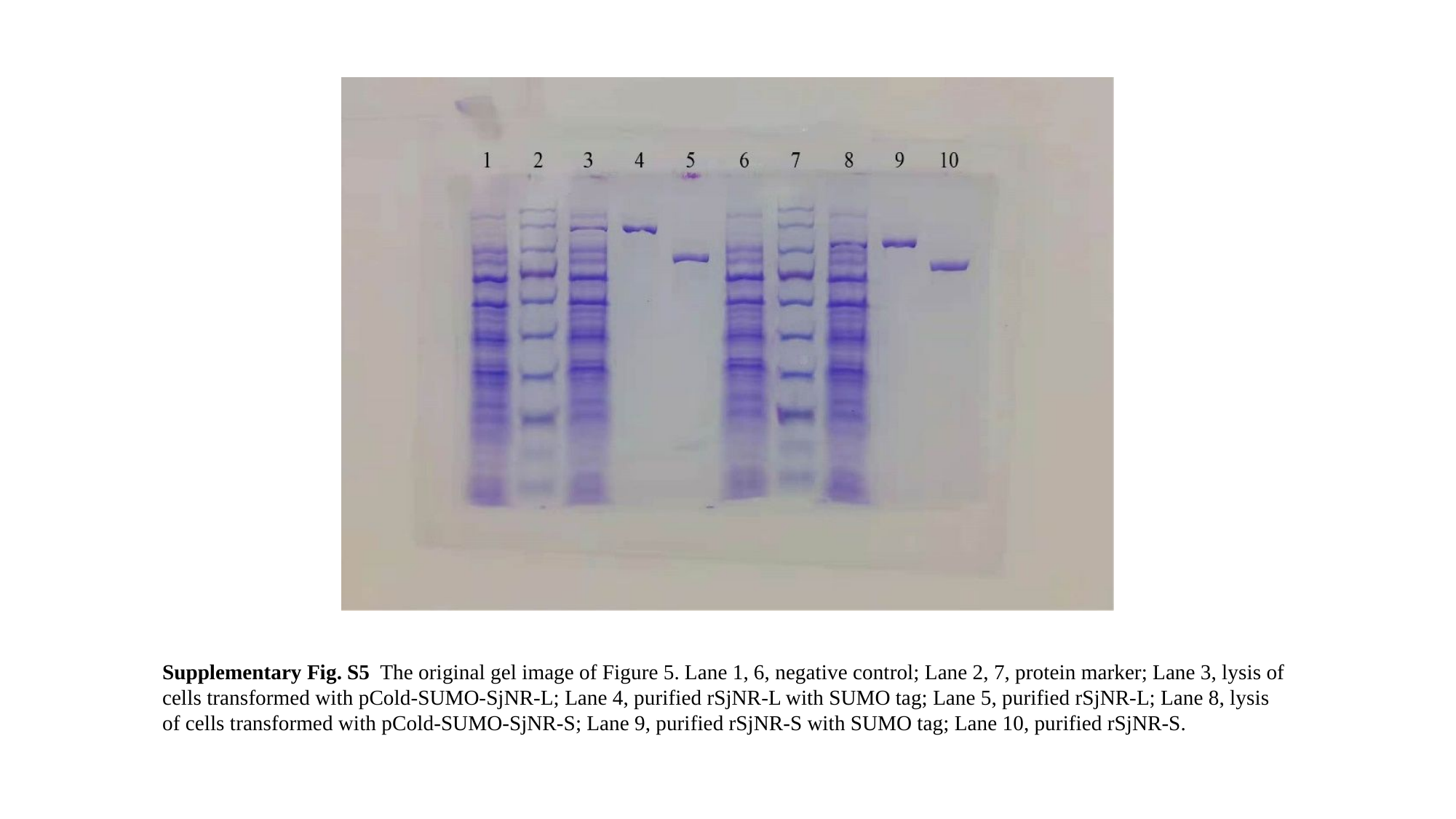

Supplementary Fig. S5 The original gel image of Figure 5. Lane 1, 6, negative control; Lane 2, 7, protein marker; Lane 3, lysis of cells transformed with pCold-SUMO-SjNR-L; Lane 4, purified rSjNR-L with SUMO tag; Lane 5, purified rSjNR-L; Lane 8, lysis of cells transformed with pCold-SUMO-SjNR-S; Lane 9, purified rSjNR-S with SUMO tag; Lane 10, purified rSjNR-S.
