# Supplementary material for: Cloning and characterization of nitrate reductase gene in kelp Saccharina japonica (Laminariales, Phaeophyta)
Source: BMC Plant Biol. 2023 Feb 6;23:78. doi: 10.1186/s12870-023-04064-7 (PMC9901164; doi:10.1186/s12870-023-04064-7)
Supplement: Supplementary file 6 — Additional file 6: Supplementary Fig. S6. The original gel image including Fig. 7A. This gel image included two separate experimental gel diagrams, and the upper half was Fig. 7A. Lane 1–3, 6–7, 13, positive transformants; Lane 4, 9, DNA maker; Lane 5, 8, negative transformants; Lane 10, 11, negative control; Lane 12, invalid amplification. [file 12870_2023_4064_MOESM6_ESM.pptx]

## Slide 1
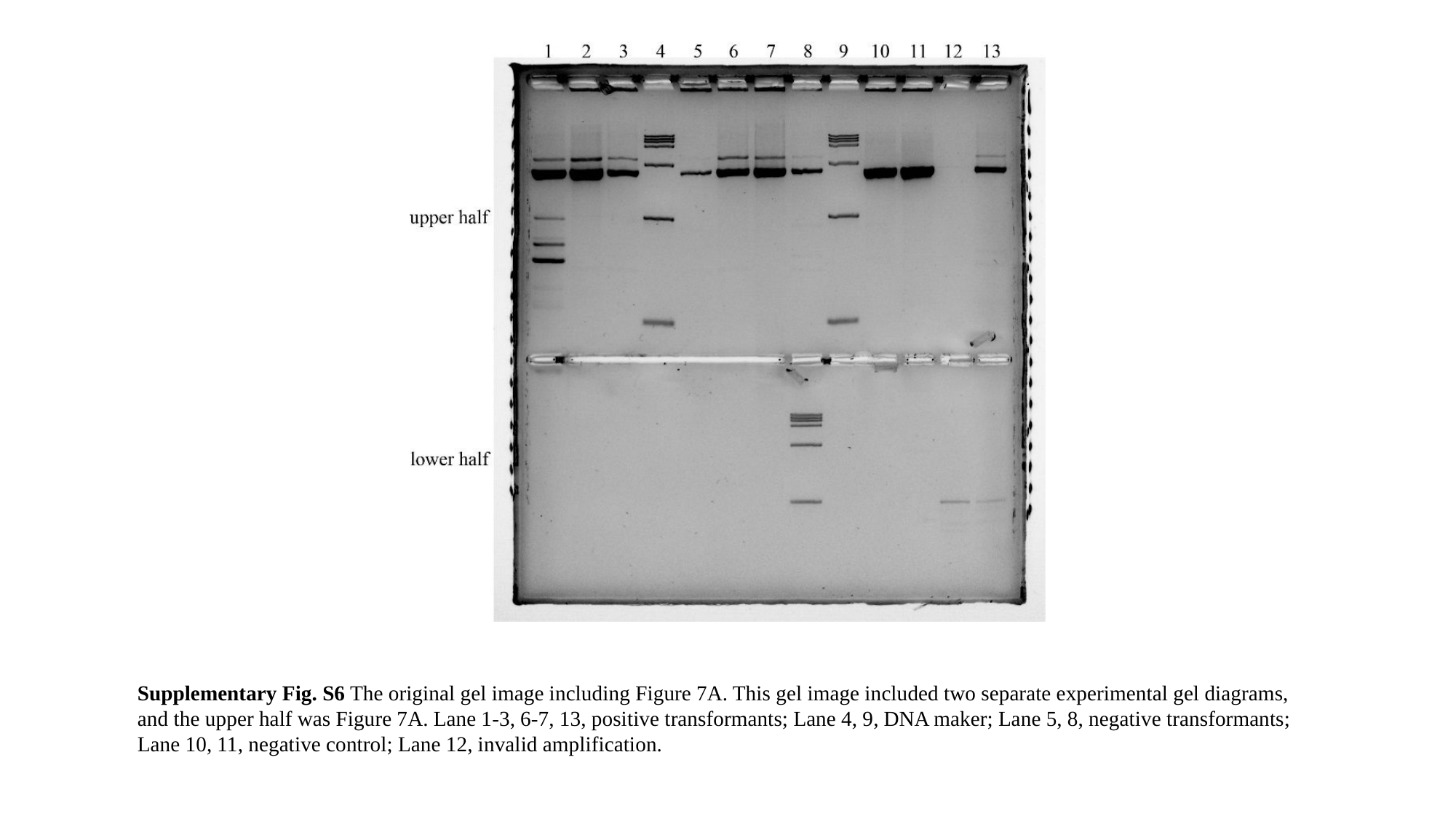

Supplementary Fig. S6 The original gel image including Figure 7A. This gel image included two separate experimental gel diagrams, and the upper half was Figure 7A. Lane 1-3, 6-7, 13, positive transformants; Lane 4, 9, DNA maker; Lane 5, 8, negative transformants; Lane 10, 11, negative control; Lane 12, invalid amplification.
